# Severe Tophaceous Gout After Decades of Progressive Disease: A Case Report

**DOI:** 10.7759/cureus.106367

**Published:** 2026-04-03

**Authors:** Grant Weiderman, Nicholas C Jianu, Skye Karastury, Puneet Mann, Nicholas Lorenz, Alexandra Jianu, Douglas Walsh

**Affiliations:** 1 Medicine, Lake Erie College of Osteopathic Medicine, Bradenton, USA; 2 Medicine, David Geffen School of Medicine at University of California, Los Angeles (UCLA), Los Angeles, USA; 3 Medicine, Kirk Kerkorian School of Medicine at University of Nevada, Las Vegas (UNLV), Las Vegas, USA; 4 Internal Medicine, MCR Health, Palmetto, USA

**Keywords:** case report, chronic gout, chronic kidney disease, functional impairment, gouty tophi, hyperuricemia, musculoskeletal disability, refractory gout, tophaceous gout, urate-lowering therapy

## Abstract

Tophaceous gout is a chronic, progressive manifestation of hyperuricemia characterized by urate crystal deposition in soft tissues and joints throughout the body. Although effective urate-lowering therapies exist, management can be challenging in patients with prior medical conditions such as renal insufficiency and chronic kidney disease. This often leads to medication intolerance with subsequent joint deformity and disability. Here, we present a 63-year-old man with severe, chronic tophaceous gout that has caused significant limitations. Despite years of treatment with various methods, this patient has yet to receive appreciable relief.

## Introduction

Gout is the most common form of inflammatory arthritis, caused by the deposition of monosodium urate crystals in the joints and soft tissues due to chronic hyperuricemia [1]. Monosodium urate crystal formation occurs when serum urate levels exceed the physiologic saturation threshold of approximately 6.8 mg/dL [1]. Globally, gout affects approximately 1%-4% of the adult population, with increasing prevalence in developed countries due to aging populations, dietary factors, and comorbid conditions such as metabolic syndrome and chronic kidney disease (CKD) [1].

While acute gout typically presents with episodic arthritis, tophaceous gout represents the chronic and severe end of the disease spectrum, characterized by subcutaneous deposits of urate crystals (tophi) that can cause joint destruction, deformity, and disability [2]. Tophi usually develop after years of untreated or poorly controlled hyperuricemia and are most often found in the ears, elbows, fingers, and toes.

Patients with CKD are at particularly high risk for tophaceous gout due to impaired renal excretion of uric acid and limited tolerance to standard urate-lowering agents [3]. The prevalence of gout is markedly higher in patients with CKD, with estimates suggesting rates exceeding 20% in advanced stages of renal disease. This bidirectional relationship further complicates management, as CKD both contributes to hyperuricemia and restricts available treatment options [3]. Despite advances in therapy, there remains a gap in the management of severe, treatment-refractory tophaceous gout in patients with advanced CKD, particularly when pharmacologic options are limited. Massive or “giant” tophi remain clinically significant because they can lead to pain, infection, and severe functional impairment, especially in patients with multiple comorbidities that restrict treatment options.

We report a case of extensive tophaceous gout in a patient with CKD stage IV and multiple systemic comorbidities, illustrating the diagnostic and therapeutic challenges in managing advanced gout when pharmacologic options are limited. This case is notable for its extreme severity, prolonged disease course spanning decades, and resistance to multiple conventional therapies, ultimately resulting in profound functional limitation and disability. Despite advances in urate-lowering therapy, severe tophaceous gout remains a significant cause of disability, particularly in patients with CKD who have limited pharmacologic options, highlighting the importance of early and sustained urate control.

## Case presentation

**Figure 1:**
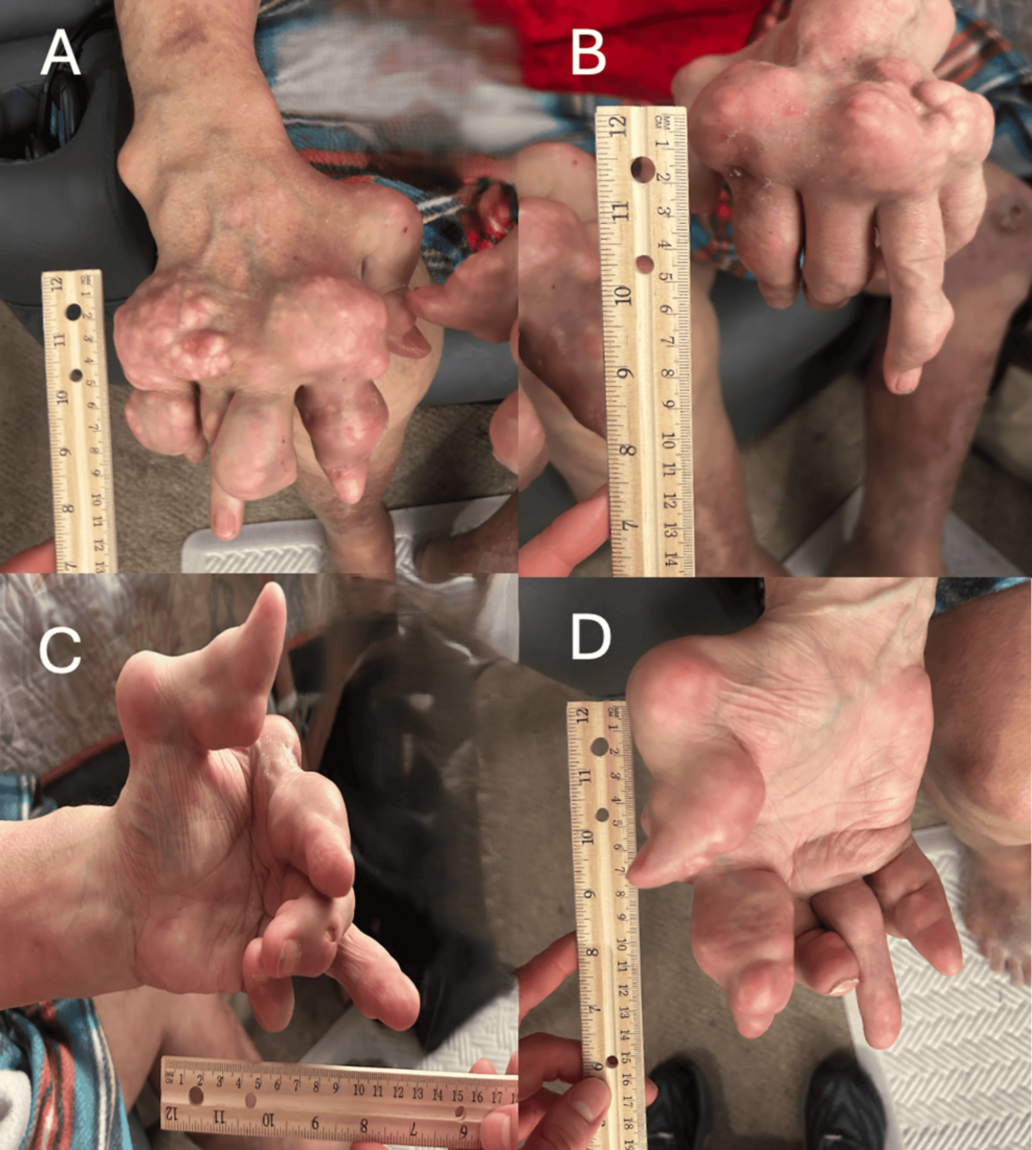
Severe tophaceous gout involving the hands (A) Dorsal view of the right hand demonstrating extensive tophaceous deposits with marked soft tissue enlargement and digital deformity. (B) Dorsal view of the left hand view highlighting the size and protrusion of tophaceous masses along the dorsal and lateral aspects of the hand. (C) Palmar view of the right hand illustrating flexion deformities and involvement of the digits with associated functional distortion. (D) Palmar view of the left hand further demonstrating the extent of tophaceous involvement and soft tissue expansion. A ruler is included in each panel for scale

**Figure 2:**
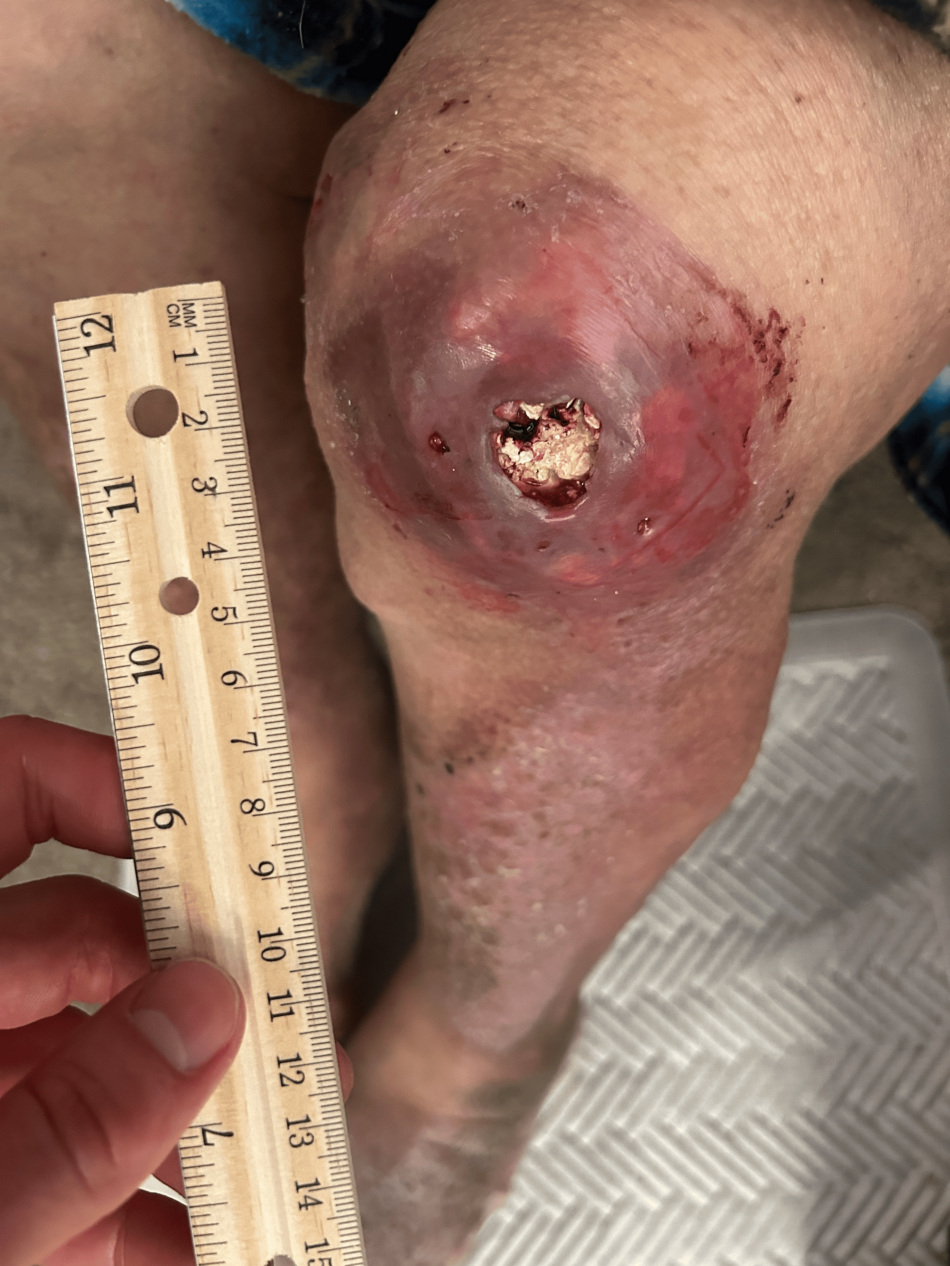
Photograph of the right knee demonstrating a large, erythematous subcutaneous tophaceous lesion over the anterior knee with central ulceration and extrusion of chalky white material consistent with monosodium urate crystal deposition. A measuring ruler is shown for size reference

**Figure 3:**
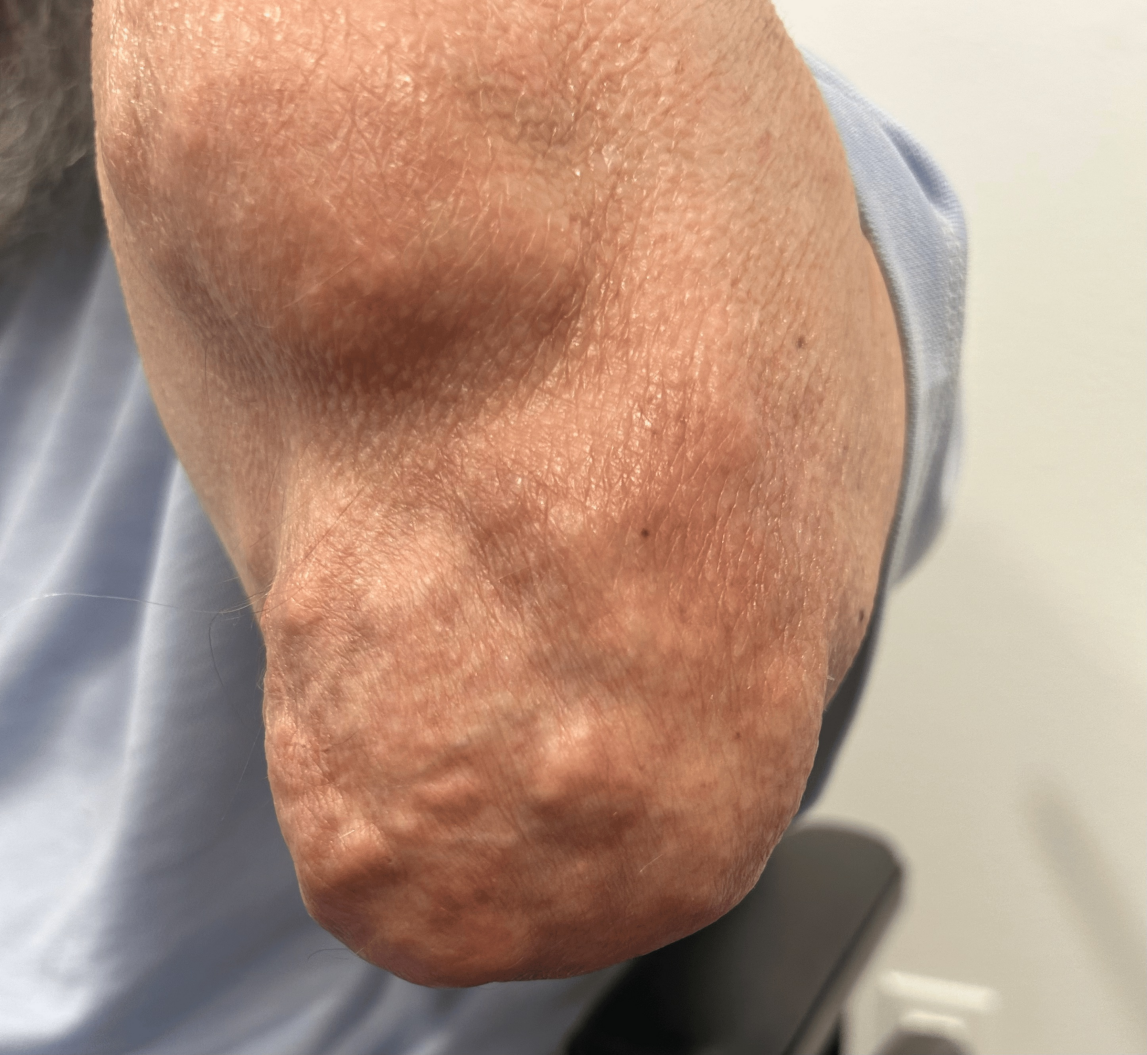
Right elbow demonstrating multiple subcutaneous nodular deposits over the olecranon region consistent with tophaceous gout, resulting in visible soft tissue enlargement and contour irregularity

A 63-year-old man with a long-standing history of chronic tophaceous gout presented with extensive nodular deformities involving both hands, elbows, and knees (Figures 1-3). He was first diagnosed with gout in 1992 and reported recurrent flares over the following three decades, with gradual enlargement of firm, chalky-white subcutaneous nodules consistent with gouty tophi. Over the subsequent years, these lesions progressively enlarged and became more numerous, ultimately resulting in severe deforming disease. These lesions ultimately produced marked joint deformity, functional limitation, and intermittent drainage with secondary staphylococcal infections. This progression led to significant impairment in activities of daily living.

His past medical history included CKD stage IV, peripheral arterial disease, avascular necrosis of the right hip, hypertension, anemia secondary to CKD, low vitamin D and testosterone, generalized anxiety disorder, chronic pain syndromes, and paroxysmal atrial fibrillation. He was also diagnosed with primary adenocarcinoma of the colon and treated with a robotic-assisted right hemicolectomy in August of 2024, and continues oncology follow-up. The patient is wheelchair-bound due to immobility from hip osteonecrosis and joint disease. The patient’s height is 5 feet 7 inches, and weight was 214 lbs, corresponding to a body mass index of 33.5 kg/m², consistent with obesity. The patient reported a prior history of alcohol use, although the quantity and duration were not clearly documented. Dietary habits were not well characterized in the available medical record. Medication adherence was uncertain based on intermittent use of prescribed therapies.

He has used allopurinol 100 mg intermittently in the past because of gastrointestinal (GI) intolerance and has also been treated with probenecid 500 mg daily and colchicine 0.6 mg daily. He recalled prior use of rofecoxib and celecoxib for pain control. Despite these therapies, he experienced progressive tophaceous deposition leading to severe deformity. His highest documented serum uric acid levels were 14.1 and 13.8 mg/dL (reference range: 3.5-7.2 mg/dL), indicating longstanding uncontrolled hyperuricemia. A rheumatology note from 2022 described consideration of pegloticase infusions with methotrexate pretreatment, even though therapy was ultimately deferred due to patient intolerance. The patient stated he has one relative with gout, but no other family history of autoimmune disease.

During the interview, the patient reported that the tophi initially felt soft and “liquid-like” but had become hard and rock-like in recent years, consistent with progressive crystal deposition over time. He described painful cracking and recurrent swelling of the nodules, with only temporary relief after manual drainage of the crystals by one physician. He noted participation in two research studies at a cancer center (in 2007 and a later trial he discontinued), but was uncertain of their details. None of his prior medications or treatments had reduced the size of the tophi, which have worsened in recent years.

On examination, there were large, firm, irregular subcutaneous nodules over the dorsal and palmar aspects of both hands with deformity of the interphalangeal joints (Figures 1-3). Additional tophi were present at the elbows and knees. The overlying skin was intact without current ulceration or drainage. These findings were associated with significant functional limitation, particularly affecting hand use and mobility, consistent with advanced tophaceous gout.

Laboratory testing in 2024 showed a serum uric acid level of 9.8 (reference range: 3.5-7.2 mg/dL), and subsequent labs in 2025 demonstrated renal function consistent with CKD IV (Table 1). Imaging was not available for review, but prior documentation described gouty arthropathy with extensive tophaceous deposits.

**Table 1:** Renal function markers, including serum creatinine, blood urea nitrogen, and eGFR, as well as hemoglobin, obtained from the patient’s most recent blood draw, with corresponding reference ranges eGFR: estimated glomerular filtration rate

Variable	Patient value	Reference range
Blood urea nitrogen	41	8-27 mg/dL
eGFR	26	>59 mL/minute/1.73 m^2^
Serum creatinine	2.64	0.76-1.27 mg/dL
Hemoglobin	9.0	3.5-17.5 g/dL

A simplified clinical timeline is as follows: initial diagnosis of gout in 1992, progressive tophus formation and recurrent flares over the next three decades, participation in research studies in 2007, consideration of pegloticase therapy in 2022, colon cancer resection in August 2024, and the most recent laboratory evaluation in 2025.

The patient’s condition was managed conservatively with allopurinol and colchicine prophylaxis, with rheumatologic follow-up for potential biologic therapy initiation. Despite medical management, his gouty tophi have continued to grow. At the time of the most recent follow-up, no surgical intervention had been pursued, and management remained focused on medical therapy given the patient’s comorbidities, treatment intolerance, and patient resistance to changing the regimen. Despite medical management, his gouty tophi have continued to grow, reflecting refractory disease despite multiple prior therapeutic attempts.

## Discussion

The case highlights an advanced presentation of chronic tophaceous gout complicated by long-standing hyperuricemia, stage IV CKD, and intolerance to standard urate-lowering therapies. Persistent hyperuricemia, as seen in this patient, leads to the deposition of monosodium urate crystals in periarticular and subcutaneous tissues, leading to tophi formation, joint deformity, and functional disability. Despite being a preventable disorder, gout remains a large cause of morbidity when control of urate is not present. This case illustrates the natural progression of inadequately controlled gout over decades, culminating in severe tophaceous burden and functional impairment. Compared to previously reported cases, this presentation represents a particularly advanced and treatment-refractory form of tophaceous gout, with extensive multijoint involvement and progression despite multiple prior therapeutic interventions, highlighting the severity of the disease in this patient.

The management of gout in patients with CKD presents significant challenges in treatment. Reduced renal clearance of uric acid exacerbates hyperuricemia and limits the functionality of uricosuric agents, including probenecid, which rely on intact renal tubular secretion for their mechanism of action [4]. Studies have shown great results with allopurinol treating patients with CKD; in a study done by Badve et al., the estimated glomerular filtration rate declined at the same rate in the allopurinol group as in the placebo group over the 104-week follow-up period, despite a reduction of 35% in serum urate levels in the allopurinol group [5]. In this patient’s case, with a GI intolerance to the medication, reintroduction of low-dose allopurinol with colchicine prophylaxis represented a pragmatic approach aimed at gradual urate reduction while minimizing adverse effects. Febuxostat, another xanthine oxidase inhibitor, represents an alternative urate-lowering therapy in patients who are unable to tolerate allopurinol [6]. Unlike uricosuric agents, febuxostat does not rely on renal excretion and may be effective in patients with moderate-to-severe CKD. According to the 2020 American College of Rheumatology guidelines, allopurinol remains the preferred first-line urate-lowering therapy, including in patients with CKD, with febuxostat serving as a reasonable alternative when intolerance occurs [6]. In this patient’s case, the use of febuxostat was not documented in the available medical record, highlighting a limitation in longitudinal data and the challenges of reconstructing prior treatment decisions in complex patients. Contemporary management strategies emphasize a treat-to-target approach with a goal serum urate level of <6 mg/dL, or <5 mg/dL in patients with tophaceous gout, although achieving these targets may be particularly difficult in patients with advanced CKD and medication intolerance [6].

In patients with refractory tophaceous gout, pegloticase, an enzyme that aids in the oxidation of uric acid to allantoin, has illustrated its efficacy in quickly reducing serum urate levels and promoting tophus resorption [7]. However, barriers such as immunogenicity and infusion reactions often limit its utilization. The patient’s refusal of pegloticase highlights a broad clinical issue; even when there are effective biologic therapies on the market, their usage will be limited by several factors outside of the practicing physician. From an analytical perspective, this case represents treatment-refractory gout driven by both pharmacologic limitations and patient-related factors, emphasizing the importance of individualized treatment strategies and shared decision-making.

Chronic tophaceous gout can be managed through medical therapy, surgical intervention, or a combination of both. Medical management alone has been shown to achieve significant improvement in select cases. For instance, a 52-year-old man with severe, bulky tophaceous gout was successfully treated with an intensive pharmacologic regimen consisting of allopurinol, colchicine, benzbromarone, and intermittent rasburicase infusions, leading to near-complete resolution of tophi over a 24-month period [8]. The concurrent use of multiple urate-lowering agents enabled aggressive serum urate reduction and dissolution of tophaceous deposits. This approach may be particularly beneficial for patients who are poor surgical candidates or who prefer to avoid operative management. In contrast, the present case demonstrates persistent progression despite multiple therapeutic strategies, further underscoring the severity and refractory nature of the disease in this patient.

In cases of massive or deforming tophaceous gout, surgical intervention may be indicated to alleviate pain, restore function, and correct joint deformity when medical management alone is insufficient. Surgical intervention is typically reserved for patients with refractory or advanced tophaceous gout who develop complications such as severe pain, functional impairment, joint destruction, nerve compression, ulceration, or recurrent infection, particularly when medical management alone is insufficient [9]. Surgical options include simple tophus excision, debridement with joint preservation, and reconstructive procedures for advanced destruction [9]. A case report by Janardhana Aithala and Shetty described a patient with bilateral metatarsophalangeal joint involvement who underwent tophus excision and soft-tissue reconstruction, resulting in marked improvement in joint mobility and relief of pain [10]. This case demonstrates that even in severe, long-standing disease, surgical excision combined with reconstruction can yield favorable functional and cosmetic outcomes, particularly when massive tophi cause ulceration, infection, or significant mechanical impairment. Surgical management is therefore an important option for patients with refractory or disabling tophaceous gout, especially when pharmacologic urate-lowering therapy is contraindicated or ineffective. Although the clinical presentation is characteristic of advanced tophaceous gout, the differential diagnosis for subcutaneous nodular lesions includes rheumatoid nodules, calcinosis cutis, tumoral calcinosis, and other crystal deposition disorders. However, the patient’s long-standing hyperuricemia, characteristic distribution of lesions, and extrusion of chalky material strongly support the diagnosis of tophaceous gout.

This case not only highlights the pharmacological limitations that are present in advanced gout but also the profound physical and psychosocial consequences. Constant pain, disfigurement, and loss of mobility have significantly impaired the patient’s quality of life. Yet, his preserved optimism and engagement with care emphasize the importance of holistic management.

## Conclusions

Advanced tophaceous gout presents significant management challenges, particularly in the setting of CKD and medication intolerance. This case demonstrates how prolonged uncontrolled hyperuricemia and intolerance to standard urate-lowering therapies can lead to severe, deforming tophaceous disease with substantial functional impairment. Early urate-lowering therapy and individualized treatment strategies are essential to prevent irreversible joint damage.

In this patient, multiple pharmacologic therapies were trialed with limited success due to intolerance and disease progression, and although advanced therapies such as pegloticase were considered, they were ultimately not pursued, highlighting the complexity of real-world treatment decision-making in medically complex patients.

In patients with refractory or deforming disease, a multidisciplinary approach that includes both medical and surgical options is necessary to restore function and improve quality of life. However, once extensive tophaceous burden has developed, treatment options may be limited and less effective, emphasizing that early recognition, aggressive urate control, and close longitudinal management are critical to preventing advanced disease.
